# Enhanced Aluminum-Ion Storage Properties of N-Doped Titanium Dioxide Electrode in Aqueous Aluminum-Ion Batteries

**DOI:** 10.3390/nano14050472

**Published:** 2024-03-05

**Authors:** Le Jian, Xibing Wu, Ruichun Li, Fangzheng Zhao, Peng Liu, Feng Wang, Daosheng Liu, Qingrong Yao, Jianqiu Deng

**Affiliations:** Guangxi Key Laboratory of Information Materials, School of Materials Science and Engineering, Guilin University of Electronic Technology, Guilin 541004, China; jl198717@163.com (L.J.); xibingwu2022@163.com (X.W.); l1534092882@163.com (R.L.); zfz513723@163.com (F.Z.); liupeng@guet.edu.cn (P.L.); wf@guet.edu.cn (F.W.); dawson@guet.edu.cn (D.L.); qingry96@guet.edu.cn (Q.Y.)

**Keywords:** aqueous aluminum-ion batteries, anode, titanium dioxide, nitrogen-doping, rate performance

## Abstract

Aqueous aluminum-ion batteries (AIBs) have great potential as devices for future large-scale energy storage systems due to the cost efficiency, environmentally friendly nature, and impressive theoretical energy density of Al. However, currently, available materials used as anodes for aqueous AIBs are scarce. In this study, a novel sol-gel method was used to synthesize nitrogen-doped titanium dioxide (N-TiO_2_) as a potential anode material for AIBs in water. The annealed N-TiO_2_ showed a high discharge capacity of 43.2 mAh g^−1^ at a current density of 3 A g^−1^. Analysis of the electrode kinetics revealed that the N-TiO_2_ anodes exhibited rapid diffusion of aluminum ions, low resistance to charge transfer, and high electronic conductivity, enabling good rate performance. The successful implementation of a nitrogen-doping strategy provides a promising approach to enhance the electrochemical characteristics of electrode materials for aqueous AIBs.

## 1. Introduction

The growing energy demand has promoted the advancement of lithium-ion batteries (LIBs) and the widespread application of commercial products. However, limited lithium resources urgently require sustainable solutions like lithium recovery and the development of high-performance and safer alternatives to LIBs [1,2,3,4]. Therefore, effective alternative rechargeable battery technologies are urgently required to leverage the earth’s more abundant elements [5]. The potential of rechargeable aluminum-ion batteries (AIBs) for grid energy storage has been explored owing to aluminum’s high theoretical mass specific capacity of 2980 mAhg^−1^ induced by exchanging three electrons during the electrochemical reaction [6,7].

Due to the fact that the standard electrode potential of Al^3+^/Al is less than that of H^+^/H_2_ (−1.68 V), the reaction between aluminum foil and acid or alkali aqueous solutions produces H_2_ [8,9,10]. Therefore, electrochemical stripping or deposition of Al in a standard aqueous solution is not possible. To ensure compatibility with the Al anode, the standard electrolyte is the ionic liquid AlCl_3_/[EMIM]Cl, which possesses a broader electrochemical window and exerts a moderate corrosive effect on the Al surface, thereby stimulating the Al striping and plating reaction [11]. These ionic liquid electrolytes, on the other hand, are not recommended for use in large-scale energy storage systems because of their exorbitant expense and possible environmental repercussions [12]. Therefore, alternative aqueous electrolytes that are non-combustible and have low toxicity are urgently required for inexpensive rechargeable AIBs [13,14]. Another significant drawback that restricts the utility of AIBs is their inadequate cathode materials, which results in a low energy density [15]. At present, the cathode materials of aqueous aluminum-ion batteries mainly include transition metal oxides (TiO_2_ [16], V_2_O_5_ [17], and MoO_3_ [18]), transition metal sulfides and selenides (Mo_6_S_8_ [19], FeS_2_ [20], Co_9_S_8_ [21], CoSe_2_ [22], and Cu_2−x_Se [23]), Prussian blue analogues (CuHCF [12], K_0.02_ Cu[Fe(CN)_6_]_0.7_ 3.7H_2_O [8], FeFe(CN)_6_ [24], and K_2_CoFe(CN)_6_ [25]), and organic materials (polyaniline [26], polyimide [27], and polypyrrole [28]). While Prussian blue analogues offer benefits such as affordability, safety, and environmental friendliness, their unsuitability for use in AABs is due to their inherently limited electrochemical capacity [29]. Frequent reports have detailed the investigation of aluminum-ion batteries constructed with carbon materials. The main method used by researchers to improve the specific capacity and rate performance of carbon materials is to regulate their various properties [30]. However, the limited specific capacity of carbon-based materials hinders their further advancement. Furthermore, it has been demonstrated by researchers that a number of conductive polymer materials are capable of storing aluminum via a redox mechanism. The reported polypyrene material with four fused aromatic rings has a reversible capacity comparable to that of graphite electrodes (>100 mAh g^−1^) [31]. Nevertheless, the limited density of active sites in conductive polymer materials using anionic energy storage limits the maximum capacity of the material [32]. Hence, it is advantageous to investigate cathode materials to achieve AIBs with superior energy density and long-term stability.

Recent research has focused on TiO_2_ due to its advantageous properties, including chemical stability, environmental tolerance, affordability, simplicity of synthesis, and high capacity [33]. The utilization of nanostructured TiO_2_ materials as electrodes for aqueous AIBs has been extensively documented. Because of its significant negative redox potential (approximately −1.20 V/−0.10 V vs. Ag/AgCl) and its considerable theoretical capacity for storage aluminum, TiO_2_ is considered to be a highly sought-after material for anode applications. The common phase structures of TiO_2_ are anatase, rutile, and brookite phases [34]. The intercalation voltage of rutile is 0.06~0.77 V, and the intercalation voltage of anatase is 0.03~0.79 V, which leads to the theoretical capacity of anatase higher than that of rutile [35]. However, the electronic conductivity of TiO_2_ with various phase structures is known to be poor, and efforts have been made to enhance it [36,37]. To improve the poor electronic conductivity of TiO_2_, Lahan et al. [38] combined TiO_2_ with highly conductive nano-silver, graphene (rGO), and carbon nanotube (CNT) materials to prepare rGO@TiO_2_, CNT@TiO_2_, and Ag@TiO_2_ composite materials, and then separately studied their aluminum-ion storage performance. The results show that ultrafast diffusion of Al^3+^ in TiO_2_ can be induced by the combination of TiO_2_ and rGO (<2 wt%). Electrochemical studies show that the rGO@TiO_2_ electrode has the best performance in 0.25 mol L^−1^ AlCl_3_ electrolyte. Ojeda et al. [39] synthesized nano-TiO_2_ with different anatase and rutile phase ratios via a two-step polyacrylic acid gel method using titanium chloride as the titanium source at room temperature. The findings indicate that the TiO_2_ nanoparticles with high specific surface area have excellent rate performance, and the larger the proportion of rutile structure in mixed-phase titanium dioxide, the higher the capacity of the material. Using a hydrothermal process, Mahdi Kazazi et al. [40] successfully synthesized titanium dioxide nanoparticles with a notable degree of crystallinity and a substantial specific surface area at current densities of 0.05 A g^−1^. Consequently, the discharge capacity achieved was 180 mAh g^−1^. A.W. Holland et al. [41] have examined the aluminum store capabilities of anatase TiO_2_ nanoparticles with two particle sizes of 5 nm and 25 nm. TiO_2_ nanoparticles with a size of 5 nm exhibited an intriguing capacity. Liu et al. [9] have also investigated the performance of anatase nano-titanium dioxide array tubes for aluminum-ion storage in 1 mol L^−1^ AlCl_3_ electrolyte. The nano-titanium dioxide array tube can be reversibly implanted with the small-radius space effect of Al^3+^. A detailed description is given in this work to clarify the aluminum storage mechanism in titanium dioxide array tubes. Electrochemical studies revealed that the titanium dioxide array tube has a discharge capacity of 75 mAh g^−1^.

Although research on TiO_2_ anodes in aqueous AIBs has made some progress, the present anode material possesses the drawbacks of limited capacity and inadequate cycle stability, which are far from meeting the application requirements of aqueous aluminum- ion batteries. Herein, a novel nitrogen-doped titanium dioxide (N-TiO_2_) was effectively produced via a simple sol-gel method and assessed as an anode for aqueous AIBs. The anode exhibits a reversible charge capacity of 43.2 mAh g^−1^ and demonstrates a consistent cycling life at 3 A g^−1^. In addition to the aluminum storage mechanism in the N-TiO_2_ anode was investigated using electrochemical testing and structural characterization techniques.

## 2. Experimental Section

### 2.1. Synthesis of N-TiO_2_

In a typical experiment, the raw material terabutyl titanate (20 mL) was put into 80 mL of absolute ethanol, then stirred for 5 min to form a homogeneous and pale yellow solution, defined as solution A. Solution B was a mixed solution composed of absolute ethanol (20 mL), deionized water (20 mL), and 3 mL concentrated HNO_3_. After solutions A and B were mixed, a pale yellow gel was formed, and the precursor powders were obtained after drying at 80 °C under vacuum conditions. Finally, the precursor powders were sintered at 450 °C for approximately 2 h in air at a heating rate of 3 °C per min. As a result of this process, N-TiO_2_ was successfully obtained.

### 2.2. Structure Characterization of Materials

The crystal structure of N-TiO_2_ was characterized by X-ray diffraction with Cu-Kα1 radiation (*λ* = 1.5406 Å, Rigaku SmartLab, Tokyo, Japan). The morphologies and microstructures of the materials were examined using field-emission scanning electron microscopy (FESEM, JSM-760FPlus, Jeol, Peabody, MA, USA) and transmission electron microscopy (TEM, Tecnai G2 F20 H-800, FEI, Hillsboro, OR, USA). The chemical composition of the materials was investigated using energy dispersive spectroscopy (EDS) on a scanning electron microscope (SEM). The X-ray photoelectron spectroscopy (XPS) examination was conducted using a Thermo Scientific (Waltham, MA, USA) Nexsa multipurpose photoelectron spectrometer equipped with an analyzer pass energy of 29.4 eV. Raman spectra were obtained from a Horiba LabRAM HR800 with a 532 nm He-Ne laser (Horiba, Kyoto, Japan). The structure of the functional groups in the samples was examined by Fourier transform infrared spectroscopy (FTIR).

### 2.3. Electrochemical Measurements

The N-TiO_2_ electrode slurry was fabricated using a combination of 80 wt% active material, 10 wt% super carbon, and 10 wt% polyvinylidene fluoride in 1-Methyl-2-pyrrole solution. Subsequently, the slurry was uniformly applied onto a titanium foil and subjected to vacuum drying at 120 °C for 12 h. The mass of the active material for a round sheet with a diameter of 10 mm was approximately 0.455 mg. The electrochemical performance of the electrodes was evaluated using a three-electrode device. The counter electrode was a platinum electrode, whereas the reference electrode was an Ag/AgCl electrode. The working electrode, on the other hand, consisted of an N-TiO_2_ electrode. The electrolyte employed was a mixed aqueous solution comprising 1M AlCl_3_. Galvanostatic charging/discharging (GCD) measurements were performed using a battery tester (CT-2001A, LAND, Wuhan, China). Cyclic voltammetry (CV) and electrochemical impedance spectroscopy (EIS) tests were conducted on an electrochemical workstation (PARSTAT MC). The frequency spanned from 0.01 Hz to 100 kHz, while the perturbation amplitude was 5 mV. Electrochemical experiments were carried out at room temperature.

## 3. Results and Discussion

### 3.1. Structure and Morphology

**Figure 1:**
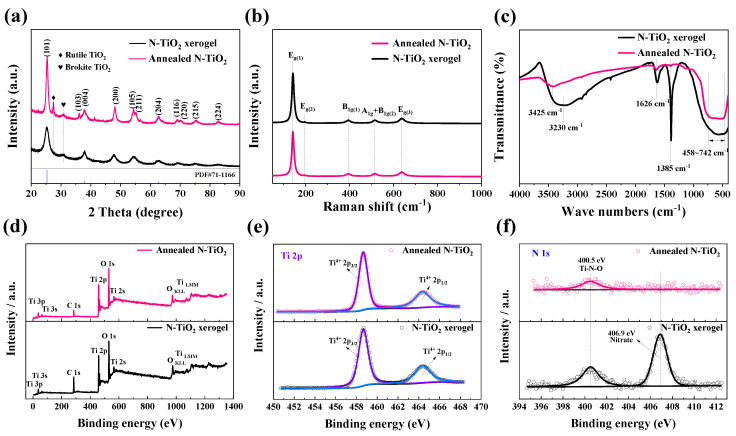
(**a**) XRD patterns of the N-TiO_2_ xerogel and annealed N-TiO_2_. (**b**) Raman and (**c**) FTIR spectra of the N-TiO_2_ xerogel and annealed N-TiO_2_. (**d**) XPS spectra and high-resolution spectra of (**e**) Ti 2p, (**f**) N 1s for the two N-TiO_2_ samples.

The synthesis of the N-TiO_2_ xerogel and annealed N-TiO_2_ was conducted according to the procedure outlined in Figure S1 (Supplementary Materials). X-ray diffraction (XRD) technique was used to analyze the crystal structure and crystallinity of the N-TiO_2_ xerogel and N-TiO_2_ annealed at 450 °C. The diffraction patterns of the N-TiO_2_ xerogel sample and N-TiO_2_ annealed at 450 °C, as shown in Figure 1a, exhibit distinct peaks at various angles. These peaks correspond to the crystal planes of anatase TiO_2_, specifically (101), (103), (004), (200), (105), (211), (204), (116), (220), (215), and (224), as identified by the (ICDD PDF No. 71-1166) [42]. Furthermore, a weak characteristic diffraction peak at 30.8° was observed for the N-TiO_2_ xerogel and annealed N-TiO_2_ materials. This characteristic peak originates from brookite-structured TiO_2_ [43,44], implying that there was a small amount of brookite TiO_2_ in the two N-TiO_2_ samples. In addition, a new XRD diffraction peak at 27.5° can be observed in the annealed N-TiO_2_ sample, which belongs to the characteristic peak of rutile TiO_2_ [45]. XRD analysis indicated that the main phase of the two N-TiO_2_ samples was anatase TiO_2_. A minute quantity of brookite TiO_2_ was detected in the N-TiO_2_ xerogel sample, whereas the annealed N-TiO_2_ sample contained minute quantities of both brookite and rutile TiO_2_. According to group theory analysis, the optical vibration mode of anatase TiO_2_ can be described as *A*_1g_(*R*) + *A*_2g_(*ia*)_g_ + *B*_1g_(*R*) + *E*_g_(*R*) + *A*_2u_(*IR*) + 2*B*_1l_(*ia*) + 3*E*_u_(*IR*) [46]. In addition to the infrared vibration mode, six Raman characteristic peaks of *E*_g(1)_, *E*_g(2)_, *B*_1g(1)_, *A*_1g_, *B*_1g(2)_, and *E*_g(3)_ can be observed in Raman spectra [47]. Figure 1b shows the Raman spectra of the N-TiO_2_ xerogel and annealed N-TiO_2_ samples. The Raman vibration mode signals at 142, 195, 394, 515, and 637 cm^−1^ of the two N-TiO_2_ samples correspond to the *E*_g(1)_, *E*_g(2)_, *B*_1g(1)_, *A*_1g_ + *B*_1g(2),_ and *E*_g(3)_ mode signals of anatase TiO_2_ [48,49]. This indicates that the two N-TiO_2_ samples possess anatase structures, which is consistent with the XRD results.

The structure of the functional groups in the samples was analyzed using FTIR, as depicted in Figure 1c. The infrared absorption signals observed in the range of 458–742 cm^−1^ were attributed to the Ti-O vibration [50,51]. The bending vibration of C-OH is commonly ascribed to the band situated at 1385 cm^−1^ [52]. The N-TiO_2_ xerogel exhibited a significantly enhanced signal compared to the annealed N-TiO_2_, mostly attributed to the presence of residual alcohols on the surface of the N-TiO_2_ xerogel specimen. The observed bands at waves of 3425 cm^−1^, 3230 cm^−1^, and 1626 cm^−1^ can be ascribed to the presence of sp^2^-coordinated graphite structured carbon. This suggests that the TiO_2_ material underwent water dissociation, leading to the formation of hydroxyl groups [52]. The N-TiO_2_ xerogel exhibited stronger infrared signals at 3230 cm^−1^ and 1626 cm^−1^ compared to the annealed N-TiO_2_ sample at 3425 cm^−1^ and 1626 cm^−1^, respectively. These results indicate that annealing considerably reduces the quantity of hydroxyl groups present on the surface of TiO_2_ [53].

Figure 1d illustrates the XPS spectra of both the N-TiO_2_ xerogel and annealed N-TiO_2_ samples. The survey XPS spectra exhibit peaks at 32.6, 58.6, 284.8, 458.8, 529.8, 560.8, 979.5, and 1102.5 eV, which correspond to the Ti, C, and O elements of N-TiO2, respectively [54]. According to the XPS results, the annealed N-TiO_2_ sample exhibited an atomic ratio of N:O:Ti of 0.83:66.34:32.82. From the Ti 2p high-resolution XPS spectra (Figure 1e), the peaks located at 458.8 eV and 464.4 eV are attributed to Ti 2p_3/2_ and Ti 2p_1/2_ bonds, respectively, suggesting the oxidation state of Ti in the two samples is +4. A discernible peak at an energy level of 400.5 eV is observed in the plotted N 1s spectra, as depicted in Figure 1f, and corresponds to the Ti-N-O chemical bond, indicating that N is successfully doped into TiO_2_ [55]. Furthermore, the presence of residual nitric acid on the surface of the N-TiO_2_ xerogel sample is confirmed by the signal peak at 406.9 eV. It can be seen that nitric acid was completely volatilized after annealing at 450 °C.

**Figure 2:**
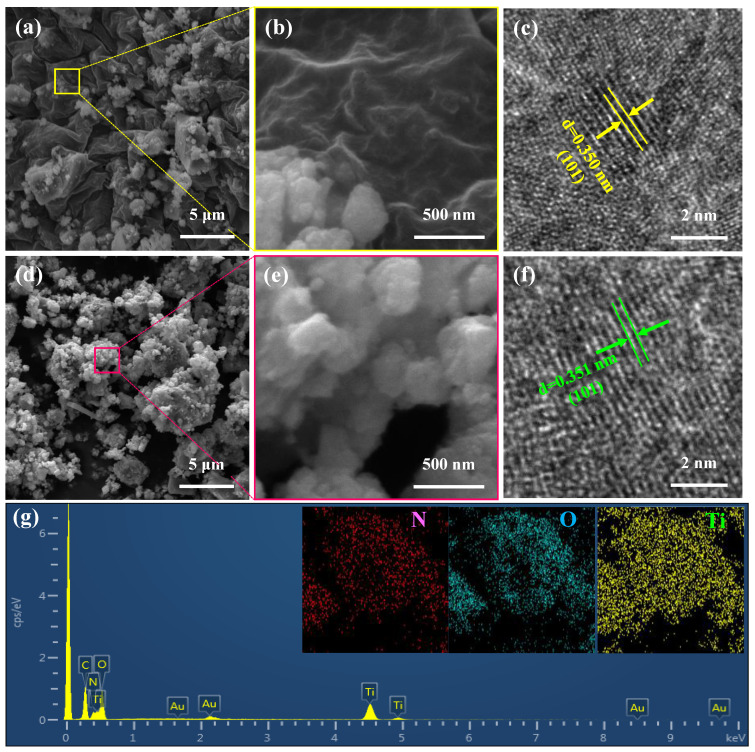
(**a**,**b**) SEM and (**c**) HRTEM images of the N-TiO_2_ xerogel. (**d**,**e**) SEM and (**f**) HRTEM images of annealed N-TiO_2_. (**g**) TEM-EDS element mapping of annealed N-TiO_2_.

The two N-TiO_2_ samples’ particle sizes and morphologies were examined using SEM (see Figure 2a,b,d,e). The SEM image (Figure 2a) reveals that the N-TiO_2_ xerogel is composed of micro-sized particles with a continuous folding morphology. After mid-temperature annealing at 450 °C, the large particles of the xerogel are broken into nano-sized particles, which helps to increase the number of reaction sites and accelerate the diffusion of aluminum ions due to its larger specific surface area and shorter pathway. Further investigation into the structural features of the two N-TiO_2_ samples was conducted using TEM and HRTEM, as shown in Figure 2c and f, respectively. The (101) crystal plane of the N-TiO_2_ xerogel can be observed in Figure 2c, and the crystal plane spacing was 0.350 nm. As shown in Figure 2f, the annealed N-TiO_2_ had higher crystallinity, and the (101) crystal plane spacing was 0.351 nm. Figure 2g shows the EDS spectrum and element mapping of the N-TiO_2_ sample annealed at 450 °C, proving that the N element was successfully doped into titanium dioxide. The EDS energy spectrum captures the electronic signal peak of the N element, providing further evidence that the N element has been effectively altered. This aligns with the results obtained from the XPS analysis.

### 3.2. Electrochemical Properties

**Figure 3:**
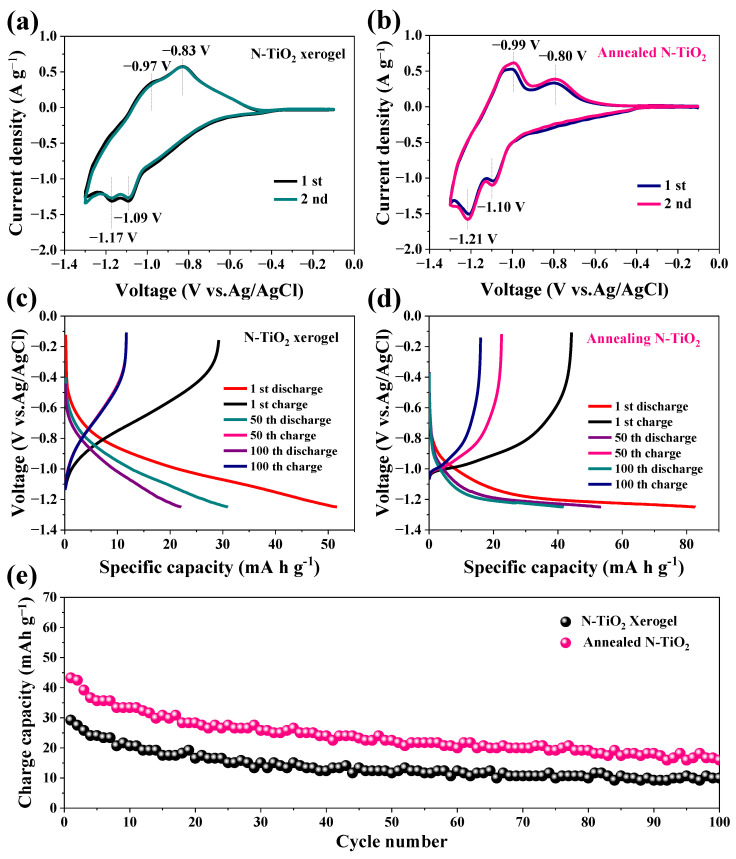
Electrochemical properties of the two N-TiO_2_ anodes. (**a**) CV curve at 9 mV s^−1^ of the N-TiO_2_ xerogel and (**b**) annealed N-TiO_2_. (**c**) The charge-discharge curves of the N-TiO_2_ xerogel and (**d**) annealed N-TiO_2_ at 3 A g^−1^. (**e**) Cycle performance.

The CV curves of the two N-TiO_2_ anodes for aqueous AIBs at a scan rate of 1 mV s^−1^ are shown in Figure 3a,b. The redox peaks at −0.83 V/−1.09 V and −0.97 V/−1.17 V are observed for the N-TiO_2_ xerogel anode in Figure 3a, indicating the two-step insertion and extraction reactions of Al^3+^ in the N-TiO_2_ is carried out in two steps. The peaks at −1.09 V and −1.17 V represent the two-step intercalation reaction of Al^3+^, and the peaks at −0.97 V and −0.83 V represent the two-step deintercalation reaction of Al^3+^. The redox peaks can be observed at −0.80 V/−1.10 V and −0.99 V/−1.21 V for the annealed N-TiO_2_ anode (Figure 3b). Compared with the N-TiO_2_ xerogel anode, the redox peak pair was more obvious, which was due to the enhanced crystallinity of the annealed N-TiO_2_.

To determine the electrochemical performance of the two N-TiO_2_ anodes, measurements of galvanostatic discharge and charge were conducted within a voltage range of −1.3 to −0.1 V. The discharge/charge curves of the N-TiO_2_ anodes are depicted in Figure 3c,d. The discharge and charge curves of the N-TiO_2_ xerogel anode (Figure 3c) do not show distinct plateau features, indicating that a portion of the aluminum storage capacity in the N-TiO_2_ xerogel anode is due to pseudocapacitive behavior. This typical slope-line feature is in line with the CV curve of the N-TiO_2_ xerogel anode. The discharge-charge curves of the annealed N-TiO_2_ anode displayed in Figure 3d present two obvious discharge/charge platforms at −0.99 V/−1.21 V. This was attributed to the annealing treatment, which improved the crystallinity of the N-TiO_2_. The discharge/charge platforms at −0.80 V/−1.10 V were not observed in the profiles because of the large polarization at high current density. The initial coulombic efficiency of the annealed N-TiO_2_ was 53%. The reason for the low coulombic efficiency was the precipitation of H_2_, irreversible reduction of Ti^4+^ to Ti^2+^ during charging, and oxidation of Ti^3+^ in the electrolyte containing dissolved O_2_. This is consistent with previous reports [7].

Figure 3e illustrates the cycle performance of the two N-TiO_2_ anodes operating at a current density of 3 A g^−1^. The annealed N-TiO_2_ anode exhibited a first reversible capacity of 43.2 mAh g^−1^. After 100 cycles, the capacity remained at 16 mAh g^−1^. The cycle performance of the annealed N-TiO_2_ anode was superior to that of the N-TiO_2_ xerogel anode because of the greater crystallinity and improved electronic conductivity of the annealed N-TiO_2_. The specific capacity of annealed N-TiO_2_ is also superior to that of previously reported materials, as shown in Table 1.

**Figure 4:**
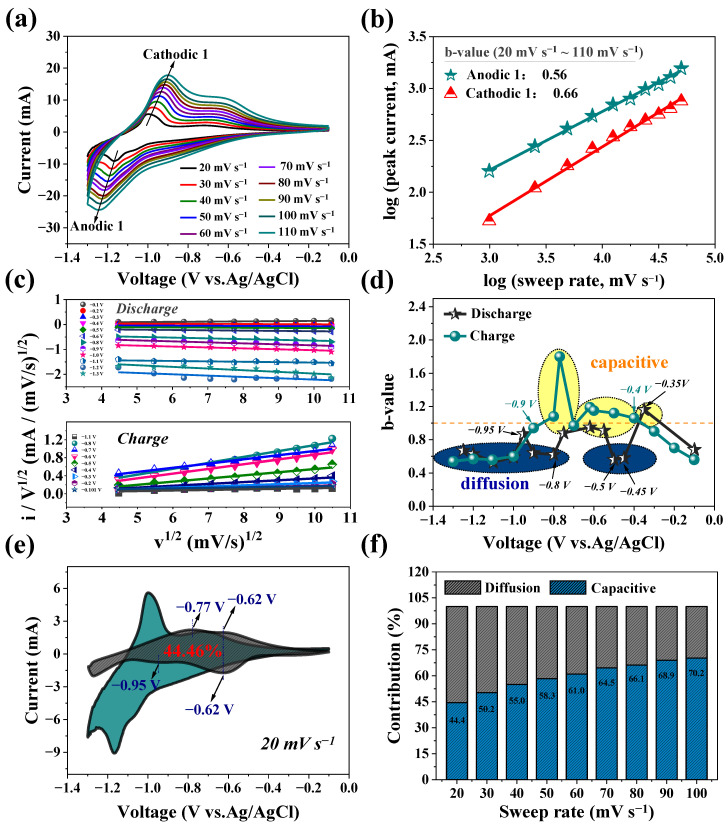
(**a**) CV curves of annealed N-TiO_2_ anodes at different sweep rates. (**b**) Correlation between log (*i*_p_) and log (*v*) values for N-TiO_2_ that has been annealed. (**c**) *b* values of annealed N-TiO_2_ at different potentials. (**d**) The plot of *i*/*v*^1/2^–*v*^1/2^ of the annealed N-TiO_2_. (**e**) Proportion of capacitance behavior (gray) and diffusion-controlled behavior (green) of annealed N-TiO_2_ at a sweep rate of 20 mV s^−1^. (**f**) The capacitance contribution of the annealed N-TiO_2_ at 20–110 mV s^−1^.

The CV technique was employed at different scan rates ranging from 20 to 110 mV s^−1^, as depicted in Figure 4a. The area-specific capacity of the CV curve originates from the total amount of charge stored by the Faraday process and non-Faraday process. The Faraday process includes capacitance behavior occurring on the surface of the electrodes and ion diffusion control behavior occurring in the bulk phase of the electrode materials. For electrochemical reactions strictly controlled by diffusion, the current value satisfies the following equation [61,62]:

(1) $$i={\mathrm{nFAC}\cdot D}^{1/2}v^{1/2}{\left(\frac{\alpha n_{\alpha }F}{\mathrm{RT}}\right)}^{1/2}\pi^{1/2}\chi^{\left(\mathrm{bt}\right)}=av^{b}\;\left(b=0.5\right)$$

For the electrochemical reaction completely controlled by the capacitance effect, the current value satisfies the following equation [63]:

(2) $$i={\mathrm{AC}}_{d}v=av\;\left(b=1.0\right)$$

Variables a and b denote adjustable parameters, while *v* represents the sweep rate. For the Faraday process with both capacitance behavior and diffusion behavior, its b value is between 0.5 and 1.0 [19]. Therefore, we can determine the Faraday process type of the electrode by analyzing the b value. To establish the correlation between the peak current (log (*ip*)) and sweep rate (log (*v*)), both variables were subjected to logarithmic transformation, as illustrated in Figure 4b. The b values of the cathode and anode peaks are calculated to be 0.66 and 0.56, respectively, indicating that the capacity of the annealed N-TiO_2_ anode is jointly controlled by the capacitance behavior and diffusion behavior during the entire discharge and charge process. The calculation of the b value at various potentials was carried out using Equation (1), as illustrated in Figure 4c. The steps involving Al-ion diffusion control are primarily observed within the potential ranges of −0.45 V to −0.50 V and −0.80 V to −1.3 V during discharge. In the remaining potential intervals, the electrochemical reaction is mainly governed by the combined control of the ion diffusion behavior and pseudo-capacitance behavior. During the charging process, the predominant step that is controlled by ion diffusion occurs within the potential range of −1.00 V to −1.30 V. On the other hand, the storage of aluminum is mostly governed by the behavior of capacitance within the potential range of −0.40 V to −0.90 V.

The proportion of capacitance behavior and ion-diffusion-controlled behavior can be estimated using the following equation [64]:

(3) $$I\left(V\right)=k_{1}v+k_{2}v^{1/2}$$

At a given potential V, *I*(V), k_1_*v*, and k_2_*v*^1/2^ represent the total current response, current caused by surface capacitive effects, and current response caused by ion diffusion-controlled behavior, respectively.

The above equation can also be reformulated as [65]

(4) $$I\left(V\right)/v^{1/2}=k_{1}v^{1/2}+k_{2}$$

By plotting *I*(V)/*v*^1/2^ and *v*^1/2^ at different potentials, as shown in Figure 4d, it is possible to determine the values of k_1_ and k_2_ from the linear relationships and then calculate the volt-ampere relationship curve of the capacitance behavior at different potentials, as shown in Figure 4e. The plot clearly demonstrates that the majority of the capacitance contribution is focused within the range of −0.4 V to −0.95 V. This observation aligns with previous estimations of the *b* value at various potentials. The capacitance contribution ratios (Figure 4f) of the annealed N-TiO_2_ anode at various sweep rates of 20 to 100 mV s^−1^ are 44.4%, 50.2%, 55.0%, 58.3%, 61.0%, 64.5%, 66.1%, 68.9%, and 70.2%, respectively.

**Figure 5:**
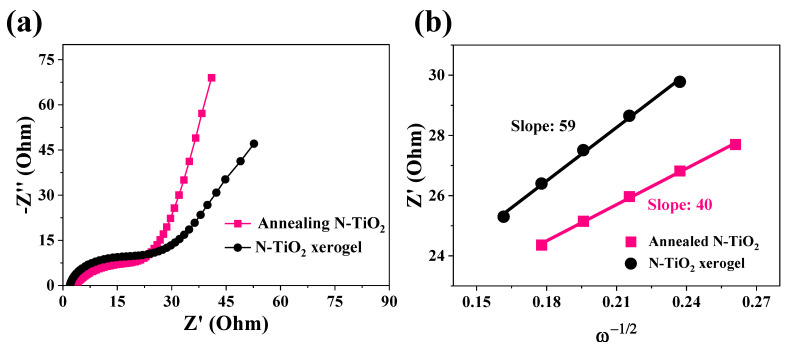
(**a**) EIS plots and corresponding (**b**) Z′–ω^−1/2^ plots of the two N-TiO_2_ anodes.

To further analyze the Al-ion kinetic behavior in the two N-TiO_2_ anodes, EIS was conducted in a frequency range of 0.01 Hz–100 kHz with an amplitude of 5 mV, as shown in Figure 5a. The plots exhibit a depressed semicircular pattern at high frequencies, which corresponds to the charge-transfer resistance (*Rct*) and capacitance (CPE1). Additionally, a sloping line is observed at low frequencies, indicating the presence of diffusion-controlled Warburg impedance (Zw) associated with Al^3+^ ions. The results demonstrate that the N-TiO_2_ xerogel anode has a higher charge transfer resistance compared to the annealed N-TiO_2_ anode. Figure 5b shows the linear curve between *ω*^−1/2^ and *Z*′. We compared the diffusion rates of Al^3+^ in the two electrodes according to the slope. The smaller the slope, the higher the diffusion rate. The observation of Figure 5b reveals that the N-TiO_2_ xerogel electrode exhibits a steeper slope, indicating a reduced diffusion rate of Al^3+^ ions in the N-TiO_2_ xerogel electrode. The annealed N-TiO_2_ electrode has a lower charge transfer resistance and higher Al^3+^ diffusion rate, suggesting better electrochemical performance.

## 4. Conclusions

In summary, novel nitrogen-doped titanium dioxide (N-TiO_2_) was successfully synthesized via a simple sol-gel technique and assessed as an anode material for AIBs. In contrast to the N-TiO_2_ xerogel, N-TiO_2_ annealed at 450 °C exhibited enhanced crystallinity, reduced particle size, and greater electronic conductivity. The annealed N-TiO_2_ anode possesses an initial reversible charge capacity of 43.2 mAh g^−1^ at 3 A g^−1^. The electrode kinetics study indicated that the Al ion storage behavior of the annealed N-TiO_2_ anode was controlled by both the surface pseudo-capacitance behavior and ion diffusion behavior. The annealed N-TiO_2_ anode had a smaller charge transfer resistance and a higher aluminum-ion diffusion rate, which indirectly proves its superior electrochemical performance.

## Figures and Tables

**Table 1:** Comparison of electrochemical properties of N-TiO_2_ and other similar materials in aqueous aluminum-ion batteries.

Electrode	Salts	Concentration	Specific Capacity (mAh g^−1^)	Capacity Retention	Refs.
TiO_2_-NTAs	AlCl_3_	1 M	75 (4 mA cm^−2^)	-	[37]
G-TiO_2_	AlCl_3_	1 M	33 (6.25 A g^−1^)	-	[56]
Ti-deficient rutile TiO_2_	NaCl, AlCl_3_	1:1	78.3 (3 A g^−1^)	-	[7]
TiO_2_	AlCl_3_, EMIMCl	1:1	40 (500 mA g^−1^)	75% (100 Cycles)	[57]
TiO_2_	AlCl_3_, KCl	1:1	15.3 (10 A g^−1^)	-	[58]
CuHCF	Al_2_(SO_4_)_3_	0.5 M	46.9 (200 mA g^−1^)	54.9% (1000 Cycles)	[59]
KNHCF	Al(OTF)_3_	5 M	46.5 (20 mA g^−1^)	52% (500 Cycles)	[60]
N-TiO_2_	AlCl_3_	1 M	43.2 (3 A g^−1^)	37% (100 Cycles)	This work

## Data Availability

Data are contained within the article.

## Supplementary Materials

The following supporting information can be downloaded at: https://www.mdpi.com/article/10.3390/nano14050472/s1. Figure S1. The schematic synthesis of the N-TiO_2_ xerogel and annealed N-TiO_2_ samples. Figure S2. UV diffuse reflectance spectra of the N-TiO_2_ xerogel and annealed N-TiO_2_. Figure S3. (a,b) High resolution XPS spectra of C 1s, O 1s for the N-TiO_2_ xerogel and annealed N-TiO_2._
