# Supplementary material for: Enhanced Aluminum-Ion Storage Properties of N-Doped Titanium Dioxide Electrode in Aqueous Aluminum-Ion Batteries
Source: Nanomaterials (Basel). 2024 Mar 5;14(5):472. doi: 10.3390/nano14050472 (PMC10934241; doi:10.3390/nano14050472)
Supplement: Supplementary file 1 [file nanomaterials-14-00472-s001.zip › nanomaterials-2792672-supplementary.pdf]

Supporting Information

Enhanced Aluminum-Ion Storage Properties of N-Doped Titanium Dioxide Electrode in Aqueous Aluminum-Ion Batteries

Le Jian, Xibing Wu, Ruichun Li, Fangzheng Zhao, Peng Liu, Feng Wang, Daosheng Liu, Qingrong Yao, Jianqiu Deng*

Guangxi Key Laboratory of Information Materials, School of Materials Science and Engineering, Guilin University of Electronic Technology, Guilin 541004, China

*Corresponding author:

E-mail:jqdeng@guet.edu.cn (Jianqiu Deng)

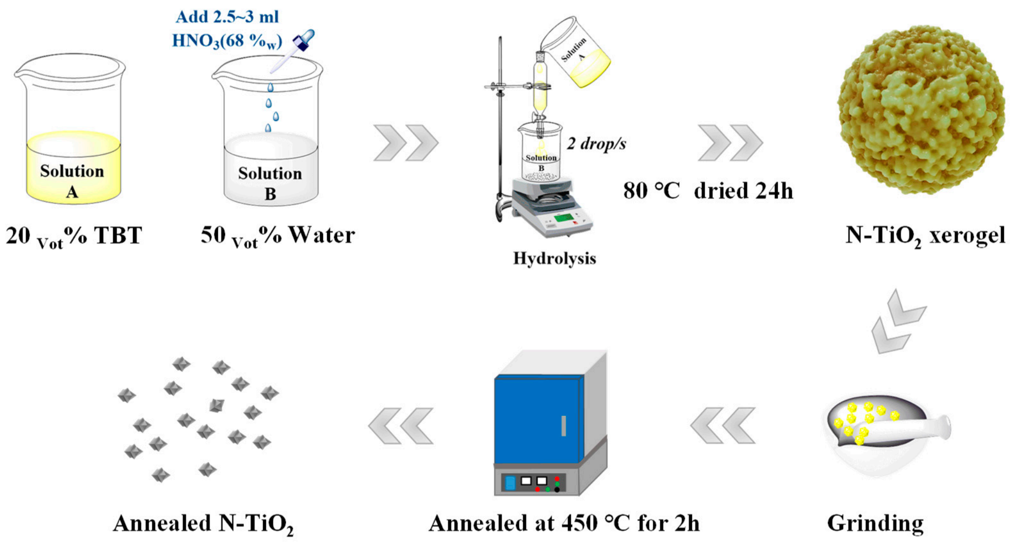

Figure S1. The schematic synthesis of the N-TiO_2 xerogel and annealed N-TiO_2 samples.

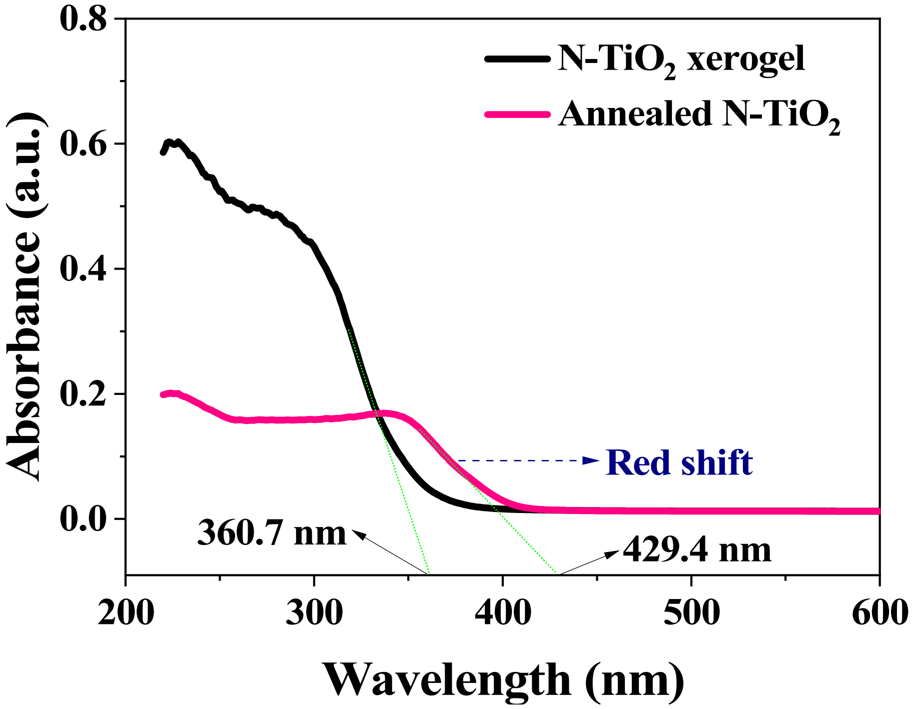

Figure S2. UV diffuse reflectance spectra of the N-TiO_2 xerogel and annealed N-TiO_2.

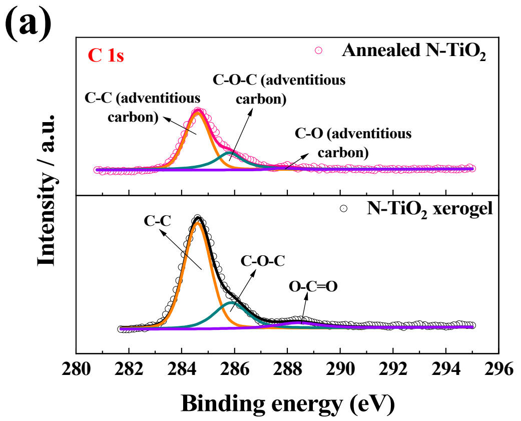

Figure S3. (a,b) High resolution XPS spectra of C 1s, O 1s for the N-TiO_2 xerogel and annealed N-TiO_2.
